# Expression of AhATL1, an ABA Transport Factor Gene from Peanut, Is Affected by Altered Memory Gene Expression Patterns and Increased Tolerance to Drought Stress in Arabidopsis

**DOI:** 10.3390/ijms22073398

**Published:** 2021-03-25

**Authors:** Ming Qin, Xiaoyan Li, Shaohua Tang, Yinglin Huang, Ling Li, Bo Hu

**Affiliations:** Guangdong Provincial Key Laboratory of Biotechnology for Plant Development, College of Life Sciences, South China Normal University, Guangzhou 510631, China; qinming@bnu.edu.cn (M.Q.); fengyuhao1@sina.com (X.L.); 13450212343@126.com (S.T.); 13268227788@126.com (Y.H.); liling502@126.com (L.L.)

**Keywords:** AhATL1, ABA, memory gene, tolerance, drought stress memory, peanut

## Abstract

*Arachis hypogaea* abscisic acid transporter like-1 (AhATL1) modulates abscisic acid (ABA) sensitivity by specifically influencing the importing of ABA into cells, and is a key player in plant stress responses. However, there is limited information on ABA transporters in crops. In this study, we found that the level of *AhATL1* expression and AhATL1 distribution increased more rapidly in the second drought (D2) compared with in the first drought (D1). Compared with the first recovery (R1), the *AhATL1* expression level and ABA content remained at a higher level during the second recovery (R2). The heterologous overexpression of *AhATL1* in *Arabidopsis* changed the expression pattern of certain memory genes and changed the post response gene type into the memory gene type. Regarding the proline and water content of Col (*Arabidopsis thaliana* L. Heynh., Col-0), *atabcg22,* and AhATL1-OX during drought training, the second drought (D2) was more severe than the first drought (D1), which was more conducive to maintaining the cell osmotic balance and resisting drought. In summary, drought stress memory resulted in a rapid increase in the *AhATL1* expression and AhATL1 distribution level, and then raised the endogenous ABA content and changed the post response gene type into the memory gene type, which enhanced the drought resistance and recovery ability.

## 1. Introduction

Plants will experience the same environmental stress many times in the process of natural growth [1]. Plants enter a “priming” state for the first stress time. When the plant faces the stress again, the response will be faster and stronger than the first time, and will store the information of the previous stress to form the stress memory [2,3]. Among the numerous environmental stresses, drought is one of the most influential and destructive [4].

Peanuts (*Arachis hypogaea.* L) are an important economic and oil crop in China. The peanut yield reduction caused by drought accounted for more than 20% of the total peanut production in China. In the actual production of peanuts, drought and water shortage environments are repeated. Plants can recognize and remember the initial stress and show more rapid and strong physiological defense when re-exposed to biotic or abiotic stress to increase their chances of survival [5]. Therefore, it is urgent to explore whether there is a drought memory in peanuts and the molecular mechanism of the peanut response to drought stress, which will provide new ideas and an alternative gene bank for improving the drought resistance of peanuts.

Abscisic acid (ABA) is not only a cell signal but also a system stress signal, which plays a key role in the plant stress resistance, in particular the drought resistance, which is reflected in the response to stress signals and the steady state of the ABA level [6]. The fine regulation of the ABA level in specific physiological processes and tissues and organs by multi-factor system coordinated regulation [7]. The ATP binding cassette (ABC) transporter family is one of the largest protein families, which can hydrolyze the energy released by ATP and complete the transmembrane transport of substrates. We further found that the rate of the ABA uptake by mutant cells was significantly lower than that of the wild type, indicating that *Arabidopsis thaliana AtABCG40/AtPDR12* is involved in the process of the ABA transmembrane transport into cells [8]. Kuromori et al. [9] also found that AtABCG25 can transport ABA across the membrane. ABCG40 and ABCG25 are involved in the absorption and efflux of ABA in plant cells, which play an important role in the rapid and effective response of plants to stress. *AhATL1* (*Arachis hypogaea abscisic acid transporter like-1*) gene was isolated and identified from Peanut in our laboratory.

Its homologous protein, AhATL1, is a member of the G subfamily of ATP binding transporters and is located in plasma membrane. *AhATL1* transcription and protein expression was up regulated by water stress and exogenous ABA treatment [10]. The study showed that during the repeated process of drought stress (dehydration–rehydration–dehydration–rehydration), the expression level of *AhATL1* was low during the first drought, and increased after rehydration, and the expression of *AhATL1* gene in leaves decreased and then increased with the duration of drought stress [11].

Currently, there are few studies on the memory regulation mechanism of plant response to drought stress, in particular, the distribution of related proteins leading to changes in plant hormone levels in this process. The biosynthesis, degradation, and inactivation of ABA affect the partial level of local active ABA, and cause further changes in ABA level through conjugation and transport. [12,13]. ABA is rapidly synthesized in root vascular parenchyma under root stress, and is then rapidly transported to leaf tissues, where it induces stomatal closure in peanut and *Arabidopsis* [14,15,16].

The compensation effect of water stress on root water absorption was positively correlated with the degree and duration of water stress before rehydration. The recovery process of photosynthesis and transpiration during rehydration showed that the compensation effect was positively correlated with the degree and duration of stress [17,18]. Our previous research found that the wilting degree of leaves in the second drought recovery (R2) was significantly lower than that in the first drought (D1). The stem was straighter, and the drought resistance was clearly improved [11]. Therefore, this treatment system can simulate the growth of the peanut memory under drought stress; however, the specific mechanism of the response to stress and memory regulation is not clear. This study will explore the changes in the AhATL1 protein and ABA levels in peanuts during the process of stress memory and provide a new perspective for the molecular mechanism of the peanut drought stress memory process.

## 2. Results

### 2.1. High Level of AhATL1 Expression and AhATL1 Distribution Resulted in Rapid Increase in ABA Content in Peanut during Drought Stress Memory

**Figure 1:**
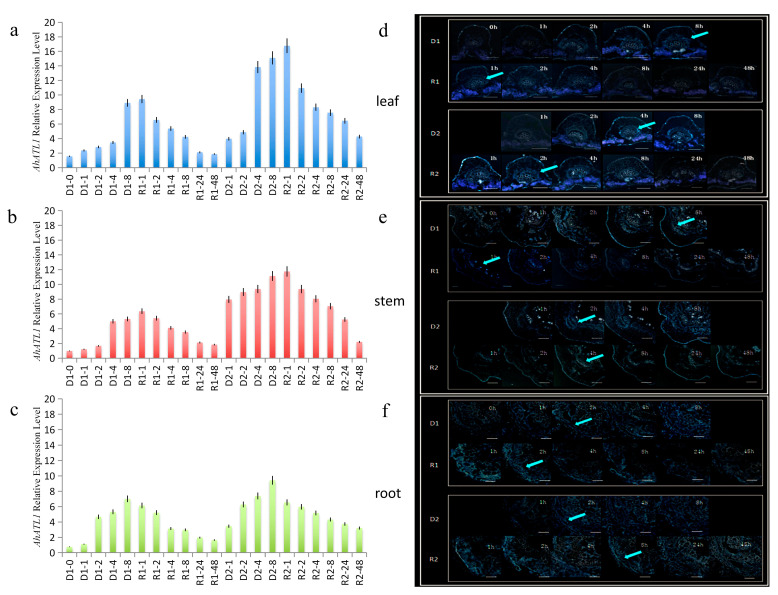
The *AhATL1* expression and AhATL1 distribution analyses in peanuts during the drought stress memory process evaluated with real-time quantitative PCR (RT-qPCR) and the immunofluorescence localization assays. The time points of the first drought (D1)-1 to the second drought recovery (R2)-48 were sampled to observe the expression and distribution-changing trend. The untreated group was used as the control (D1-0). (**a**–**c**) Expression analyses of *AhATL1* in peanut leaves, stems, and roots during the drought stress memory process. UBQ10 was used as an internal control. The mean and SD (standard deviation) were obtained from more than three biological replicates. (**d**–**f**) AhATL1 immunostaining of the leaves, stems, and roots following the same treatment regime described for a. The control samples were treated with PBS; blue fluorescence (arrows) indicates the presence of AhATL1; and no color indicates a lack of staining.

In the process of drought stress memory acquisition, the expression level of *AhATL1* in peanut leaves was increased slowly in the D1 process and increased sharply at 8 h. Compared with that in D1, *AhATL1* expression level at 4 h (in D2) was higher than that in D1 process (the same drought time node was compared). At 4–48 h of the R2 process, the expression trend of the gene was the same as that of R1, which first increased and then gradually decreased to a low level. However, the overall expression level of the R2 process was higher than that of R1 (Figure 1a). Then, we further studied the distribution level of AhATL1 and found that, under normal conditions, the specific distribution level of the AhATL1 protein in leaves was very low.

However, in the D2 process, the AhATL1 distribution increased significantly at 4 h, faster than that in D1 (8 h). During the process of R1, the AhATL1 distribution level was maintained at a low level for 4 h. In the R2 process, the AhATL1 distribution level was increased first at 2 h, and then decreased gradually (Figure 1d). The distribution of AhATL1 in R2 was stronger than that in R1 (the same drought time mode was compared).

In stems, the expression level of *AhATL1* in D1 increased remarkably at 4 h, while that in D2 increased at 1 h. During the process of R1, the expression level of *AhATL1* increased slightly at 1–2 h and then decreased gradually; R2 increased at 4 h and then decreased gradually (Figure 1b). For the AhATL1 distribution level, those in the vascular tissue of the phloem of the stems of the D1 and D2 processes were increased gradually at 2 h. At the R1 and R2 stages of rehydration, the overall distribution level of AhATL1 was low. Among them, the AhATL1 distribution level was increased a little at 1–2 h, and then gradually decreased in R1 (Figure 1e), while the AhATL1 distribution level was increased at first (to 4 h).

In the root, the expression level of *AhATL1* increased significantly at 2 h in the D1 process; however, the increasing trend became gradually flat. Meanwhile, in D2, the *AhATL1* level gradually increased at 2–8 h, and was maintained at a high level. In the process of R1, the expression level of *AhATL1* gradually decreased during the whole rehydration process, while R2 maintained a high level (1–4 h), and then gradually decreased (Figure 1c). At the protein level, the AhATL1 distribution level increased slowly and reached a higher level at 8 h in D1 and, in the D2 process, gradually increased at 2–8 h and maintained a higher level. At the R1 and R2 of rehydration, the AhATL1 distribution level decreased gradually in the whole rehydration process during the R1 process, while R2 maintained a high level (1–4 h) at first and then decreased gradually (Figure 1f).

**Figure 2:**
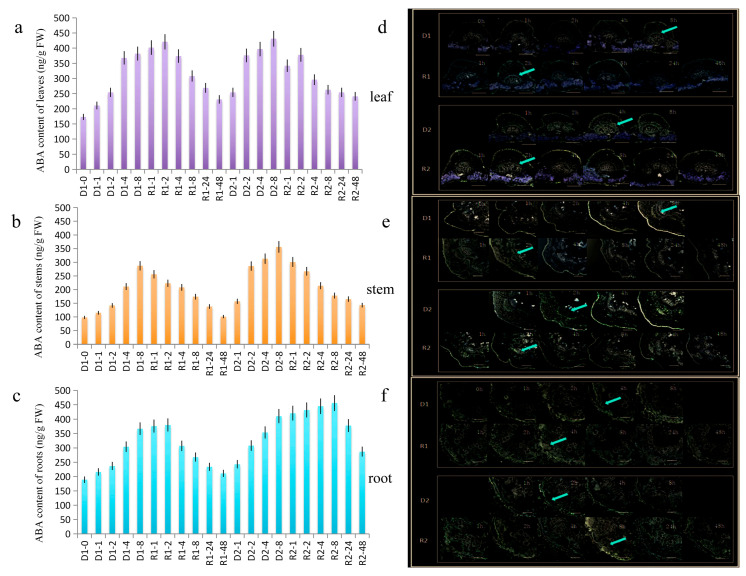
The abscisic acid (ABA) content and distribution analyses in peanut during drought stress memory process using HPLC and immunofluorescence localization assays. The time points of D1-1 to R2-48 were sampled to observe the expression and distribution-changing trends. The untreated group was used as the control (D1-0). (**a**–**c**) The ABA content of the leaves, stems, and roots in peanuts during the drought stress memory process. The mean and SD were obtained from more than three biological replicates. (**d**–**f**) The ABA immunostaining of leaves, stems, and roots following the same treatment regime described for a. The control samples were treated with PBS; green fluorescence (arrows) indicates the presence of ABA; and no color indicates a lack of staining. Bars = 50 μm for leaf panels and 100 μm for the stem and root panels.

In response to drought stress in peanuts, the expression of AhATL1 specifically affects ABA signal. We investigated the distribution and content of ABA in peanuts during the drought stress memory process. The content of ABA in peanut leaves increased continuously in the process of D1 and increased sharply at 4 h, which was 112.7% higher than at 0 h. With the drought treatment, the content of ABA increased slightly and then decreased after 4 h in R1 (Figure 2a). During D1 and D2, the distribution level of ABA showed a trend of increasing gradually, and the increasing speed of the ABA distribution level in the D2 process was faster than in the D1 process, which had obvious enhancement at 4 h (Figure 2d).

For the stem of peanuts, in the process of D1, the content of ABA increased gradually at 48 h. The ABA content was increased significantly at 2 h and then continued to increase in D2. The content of ABA maintained a decreased trend in R1, while the ABA content was increased (to 2 h) and then gradually decreased in R2 (Figure 2b). On the other hand, the distribution level of ABA equably increased with the stress in D1, while it was increased significantly at 2 h in D2. In the process of R1 and R2, the ABA distribution level increased slightly at first and then decreased gradually after 2 h; however, the overall ABA distribution level was stronger than that of R1 (Figure 2e).

In the roots, the increasing speed of the ABA content in D2 was faster than in D1, which was obviously enhanced at 2 h, while that in D1 was 4 h. In the process of R1, ABA content increased gradually at 4 h, while in the process of R2, it increased gradually at 8 h. The content of ABA increased first and then decreased in both processes (Figure 2c). For the level of the ABA distribution, those were increased in D2 faster than that in D1 and increased significantly at 2 h, and in D1 at 4 h. During the rehydration process, the ABA-specific distribution of R2 was stronger than that of R1 (Figure 2f).

### 2.2. Heterologous Overexpression of AhATL1 Alters the Expression Patterns of Some Memory Genes in Arabidopsis Thaliana

Ding et al. [19] proposed the standard of transcriptional memory in *Zea mays* and *Arabidopsis thaliana*, and believed that the transcriptional response must be different under similar stress conditions. Therefore, in the repeated dehydration stress/rehydration cycle, three types of genes were found: memory genes [+/+], [−/−], [−/+], and [+/−]; non-memory genes [+/=] and [−/=]; and post response genes [=/+] and [=/−]. The first symbol indicates that the transcriptional level of plants under the first stress was higher (+) or lower (−) than that under normal watering. The second symbol indicates that the transcriptional level of the second stress was higher (+) or lower (−) than that of the first stress. Non-memory genes [+/=] and [−/=] indicated that the transcriptional levels up or down regulated in the first stress were similar to those in the first stress. The post response genes [=/+] and [=/−] indicated that the transcriptional levels were similar in the first stress, but up-regulated or down-regulated in the second stress. In the process of rehydration, the genes that produced a similar level of transcriptional response to each stress are considered non-memory genes, and the genes showing significant differences from the first stress response in the subsequent stress are considered memory genes.

To further explore the response mechanism of *AhATL1*-mediated drought stress memory, *Arabidopsis thaliana* was used. After several short-term drought treatments, compared with the control group, the leaf cells of the experimental group in *Arabidopsis* plants had stronger water holding capacity and higher expression level of drought response genes. These results indicate that stress response genes can be “trained” by stress and show differential expression in response to repeated stress [19]. Therefore, plants can distinguish between single stress and repeated stress, and regulate the expression of stress response genes accordingly. In Arabidopsis and maize, after repeated drought stress, memory genes with functions in ABA and JA regulatory pathways were found by RNA SEQ analysis, which indicated that plant hormones were involved in short-term drought stress memory [19,20,21]. Drought stress can induce many signaling pathways [14]. Therefore, we will focus on the response to drought stress and ABA signaling pathway-related genes. These include: stress response marker gene, *AtRD29A*; ABA synthesis gene, *AtNCED3* (9-cis-carotenoid dioxygenase gene); ABA degradation gene, *AtCYP707A1* (cytochrome P450 monooxygenase gene); transformation of free ABA into binding ABA gene, *AtUGT71C5* (ABA glycosyltransferase gene); the binding ABA was transformed into free ABA gene, *AtBG2* (β—glucosidase gene); the downstream genes of ABA signaling pathway and regulation: *AtPP2C5* (protein phosphatase gene), *AtOST1* (atsnrk2.6, protein kinase gene), *AtABI5* (ABA response binding factor), *AtAREB1* (ABA response binding factor), *AtSLAC1* (plasma membrane located anion channel gene), *AtALMT12* (plasma membrane located anion channel gene); ABA transport genes: *AtABCG11* (wax export gene), *AtABCG22* (ABA input gene), *AtABCG40* (ABA input gene), and *AtABCG25* (ABA output gene).

**Figure 3:**
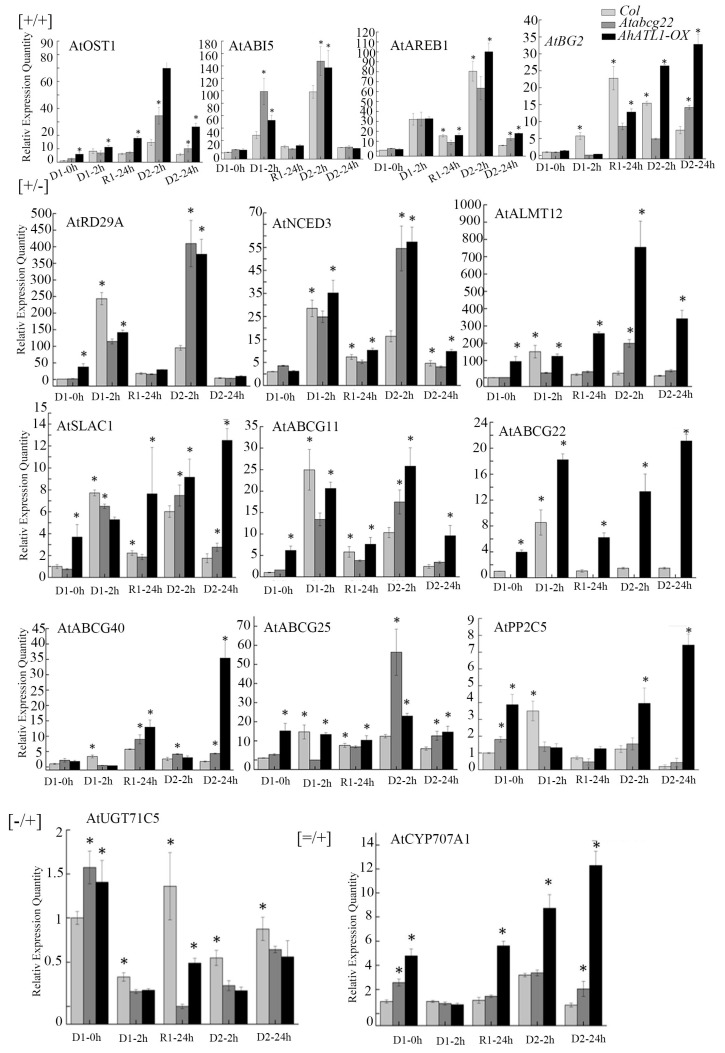
Changes in the expression of ABA and stress response related genes under drought training. [+/+], [+/−], [−/+], and [=/+] indicate the expression patterns of each gene in wild-type Col. ‘*’ Indicates a significant difference between AhATL1-OX, *Atabcg22*, and Col plants under the same dehydration and rehydration treatment conditions (*p* < 0.05). Asterisks indicate significant differences from Col in D1 0h (Student’s *t* test *p* values, * *p* < 0.05).

We found that *AtRD29A*, *AtNCED3*, *AtPP2C5*, *AtSLAC1*, *AtALMT12*, *AtABCG11*, *AtABCG22*, *AtABCG40,* and *AtABCG25* were memory gene types with [+/−] expression patterns, indicating that these genes were induced in the D1 process and down-regulated in the D2 process. *AtUGT71C5* is a memory gene type, and its expression pattern is [−/+], indicating that the gene is down-regulated in the D1 process and up-regulated in the D2 process. *AtCYP707A1* is a post response gene type with a [=/+] expression pattern, which indicates that the expression of *AtCYP707A1* in the D1 process is similar to that in the D1-0 h process, and this is further induced in the D2 process (Figure 3). There were two types of gene expression change in Col *Arabidopsis* plants, including six expression patterns (Table 1). *AtOST1*, *AtABI5*, *AtAREB1,* and *AtBG2* were memory gene types, which were [+/+] expression patterns, which indicated that these genes were induced to express in the D1 process and to a higher expression level in the D2 process.

On this basis, we found that *AtOST1*, *AtABI5,* and *AtAREB1* were memory gene types and [+/+] expression patterns, while *AtBG2* changed from the [+/+] expression mode to the [−/+] expression mode, which was a memory gene type in *AhATL1-OX* and *atabcg22* plants. The memory gene expression patterns of *AtRD29A*, *AtNCED3*, *AtSLAC1*, *AtALMT12,* and *AtABCG11* changed from [+/−] to [+/+]. The gene expression patterns of *AtPP2C5*, *AtABCG40,* and *AtABCG25* changed from [+/−] to [−/+], which were memory gene types. The gene expression pattern of *AtABCG22* was [+/−], which was the same as Col, while in the *atabcg22* mutant, the gene expression pattern of *AtUGT71C5* was [−/+], which was the same as Col. The gene expression pattern of *AtCYP707A1* changed from [=/+] to [−/+], and the gene type changed from the post response gene to a memory gene (Table 1, Figure 3). These results indicate that the gene expression pattern of the *AhATL1-OX* strain was similar to that of the *atabcg22* strain, but different from Col. Compared with Col, heterologous overexpression of *AhATL1* in Arabidopsis changes the expression pattern of certain memory genes as well as the post response gene type into the memory gene type.

[+/+], [+/−], [−/+], and [=/+] indicate the expression patterns of each gene in wild-type Col. ‘*’ Indicates a significant difference between AhATL1-OX, *atabcg22*, and Col plants under the same dehydration and rehydration treatment conditions (*p* < 0.05). Asterisks indicate significant differences from Col in D1 0h (Student’s *t* test *p* values, * *p* < 0.05).

### 2.3. Overexpression of AhATL1 in Arabidopsis Enhances Tolerance Ability in the Drought Stress Memory Process

**Figure 4:**
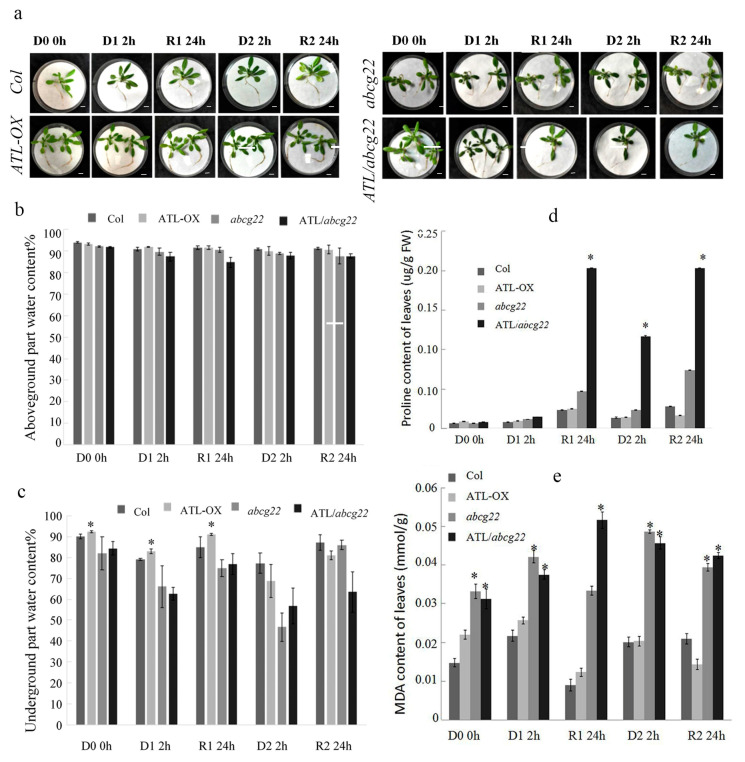
Overexpression of *AhATL1* in *Arabidopsis* enhanced the plant tolerance during the drought stress memory process. (**a**) Drought tolerance phenotypes of four lines (Col, *P35S::AhATL1-eGFP*, *atabcg22,* and *P35S::AhATL1-eGFP/atabcg22*) that were dehydrated for 2 h and rehydrated for 24 h two times. (**b**) The aboveground water content of the four lines that were dehydrated for 2 h and rehydrated for 24 h two times. (**c**) The belowground water content of the four lines that were dehydrated for 2 h and rehydrated for 24 h two times. (**d**) The proline content of the four lines that were dehydrated for 2 h and rehydrated for 24 h two times. (**e**) The MDA content of the four lines that were dehydrated for 2 h and rehydrated for 24 h two times. All experiments, means, and SDs were obtained from more than three biological replicates. ‘*’ indicates a significant difference between AhATL1-OX, *atabcg22*, *P35S::AhATL1-eGFP/atabcg2*2, and Col plants under the same dehydration and rehydration treatment conditions (*p* < 0.05). Asterisks in (**b**) to (**e**) indicate significant differences from Col in D1 0h (Student’s *t* test *p* values, * *p* < 0.05).

In order to further verify whether the expression changes of memory related genes under drought stress cause the changes in physiological indexes and plant phenotype, we conducted the following studies. ABA transporter ABCG22 has the function of ABA input, which is located on the plasma membrane of guard cells. The expression of *AtABCG22* gene is induced by ABA, and the stomatal closure of *atabcg22* mutant decreases under the conditions of low air humidity, high CO_2_ or exogenous ABA, indicating that it regulates ABA transport and may enhance ABA inflow into guard cells [9,20]. Therefore, since *AtABCG22* is a homologous gene in *Arabidopsis thaliana*, which has ABA transport function and affects drought resistance, in order to study the function of AhATL1, *atabcg22* mutant was used to observe whether it could complement each other. The localization of AhATL1—eGFP protein in cell membrane conforms to the characteristics of its membrane protein, which is consistent with the reported *AtABCG22*. Functional complementation experiments have also proved that AhATL1-eGFP has a function [10]. *AhATL1* overexpression vector *P35s::AhATL1-eGFP* was transformed into Col wild type *Arabidopsis thaliana*. Compared with Col and *P35S::AhATL1-eGFP*, *atabcg22* and *P35S::AhATL1-eGFP/atabcg22* had a darker color and more developed roots. The petioles of plants were drooped, and the leaves were curled and wilted in the D1 process, then gradually recovered after rehydration. After the second dehydration stress (D2), the rate of petiole drooping and leaf wilting was slower. The wilting degree of Col and *atabcg22* under dehydrated stress was more severe than that of *P35S::AhATL1-eGFP* and *P35S::AhATL1-eGFP/atabcg22*. After rehydration, the plants of Col, *P35S::AhATL1-eGFP* and *P35S::AhATL1-eGFP/atabcg22* recovered well, while *atabcg22* recovered poorly (Figure 4a).

After the first dehydrated stress (D1), the aboveground water content of the four lines decreased by 3.21%, 1.28%, 2.58%, and 4.80%, respectively, and changed little in the process of R1 and D2. The aboveground water content of Col and *P35S::AhATL1-eGFP* recovered to 91.03% and 90.55%, respectively, while *atabcg22* and *P35S::AhATL1-eGFP/atabcg2*2 did not recover and decreased to 87.53% and 87.49% after the second rehydration, respectively (R2) (Figure 4b). For the belowground part of the water content after D1, Col and *P35S::AhATL1-eGFP* decreased by 12.34% and 10.15%, and then that of *P35S::AhATL1-eGFP/atabcg22* decreased significantly more than *atabcg22*, which decreased by 25.82% and 19.42%, respectively, After the first rehydration (R1), the recovery degrees of the belowground water contents of *P35S::AhATL1-eGFP* and *P35S::AhATL1-eGFP/atabcg22* were greater than those of Col and *atabcg22*. Combined with the above ground water content, *P35S::AhATL1-eGFP* and *P35S::AhATL1-/atabcg22*, the plants that overexpressed *AhATL1* may tend to distribute water to the belowground when rewatered. After the second dehydrated stress (D2), the belowground water content of *atabcg22* was significantly greater than that of *P35S::AhATL1-eGFP/atabcg22*; compared with the first dehydrated stress, the belowground water content of the four *Arabidopsis* species decreased by 2.22%, 17.14%, 29.36%, and 9.30%, respectively (Figure 4c).

Proline is a kind of osmotic adjustment substance in plant cytoplasm. Under stress, the content of proline in plant increases significantly. The proline content in plants reflects the stress resistance of plants to a certain extent [22,23]. Four different *Arabidopsis* genotypes plants accumulated more proline, among which the proline content of *P35S::AhATL1-eGFP/atabcg22* was the highest and was four times higher than that of *atabcg22* after R1. Then, the proline content of the four lines (Col, *P35S::AhATL1-eGFP*, *atabcg22,* and *P35S::AhATL1-eGFP/atabcg22*) increased by 69.17%, 47.17%, 100.42%, and 681.36%, respectively, after D2 (Figure 4d).

The cell membrane was damaged by dehydrated stress, and the membrane lipid peroxidation produced malondialdehyde (MDA). After D1, the MDA contents of the four lines were increased by 46.95%, 16.72%, 27.01%, and 20.13%, respectively. During the first rehydration treatment (R1), the MDA contents of Col, *P35S::AhATL1-eGFP*, and *atabcg22* plants were decreased, which were lower than that in D1. It indirectly indicated that the membrane damage was repaired to a certain extent after rehydration, while in D2, the MDA contents of *Col*, *P35S::AhATL1-eGFP,* and *atabcg22* were increased, and those of *P35S::AhATL1-eGFP/atabcg22* were decreased. Compared with the first dehydrated stress, the MDA content of *Col* and *P35S::AhATL1-eGFP* were decreased by 7.33% and 20.87%, the cell membrane damage was lighter, and the drought resistance was enhanced. After R2, the MDA content of *P35S::AhATL1-eGFP*, *atabcg22*, and *P35S::AhATL1-eGFP/atabcg22* were decreased, during which the MDA content of the Col plants continued to accumulate (Figure 4e).

## 3. Discussion

During the growth cycle, plants will experience repeated dehydration stress, and there will be an intermittent water recovery period [19]. After repeated stress, pre-exposure to environmental stress will change the response to subsequent stresses [24,25,26,27], and the phenotypic plasticity during this process may improve the long-term survival rate and yield of plants [7,28]. Peanuts are an important economic and oil crop in China. The reduction in the peanut yield caused by drought accounted for more than 20% of the total peanut production in China. Research proved that drought training at the seedling stage could increase the yield of peanuts [14].

Crops can respond to the same stress more quickly and effectively through morphological adaptation, physiological, and hormonal changes and transcriptional modification, for example, by improving the transport and distribution of ABA, an important stress hormone, we found that both the AhATL1 protein and ABA existed in the roots, stems, and leaves of peanut plants during the process of drought stress memory.

The distribution level of the AhATL1 protein and ABA increased more rapidly and increased more rapidly in the second drought compared with in the first drought stress. In the process of rehydration, the distribution of the AhATL1 protein and ABA increased for a short time, and then decreased gradually. Compared with the first recovery, the distribution level of the AhATL1 protein and ABA remained at a higher level during the second recovery. The results show that the drought resistance ability of peanuts was enhanced after the first drought stress training.

ABA plays an important role in closing the stomata and reducing water transpiration in plants under drought stress [29]. Researchers found that the distribution level of ABA in a vascular bundle gradually increased during drought stress, and the distribution level of drought resistant peanuts was higher than that of drought sensitive peanuts [30]. For the site of ABA synthesis, previous research in the laboratory found that under drought stress, ABA in the leaves of peanut plants was mainly distributed in vascular tissue [31], which is also consistent with this point in this paper.

In addition, we also observed the ABA distribution in plant vascular tissues during the response of peanut leaves, roots, and stems to stress. After repeated drought stress on four leaf stage peanut seedlings, the leaf wilting degree, stem bending degree, and water content were compared. We found that the relative water content of the D2 peanut plants in the second drought was lower than that in the first drought, while the leaf wilting degree and stem bending degree of the peanut plants were lower than those of the first drought, and the recovery degree of leaves and stems was stronger than that of R1.

The drought resistance of peanut plants was enhanced after one drought and rehydration. This is possibly caused by the stomatal opening of peanut plants being less than in D1, suggesting that peanut plants had drought stress memory. The stomatal opening did not increase significantly, which promoted the rapid response of leaves, regulating stomatal closure, reducing transpiration, and maintaining the water content at D2 [11]. Therefore, peanut demonstrated a memory in the process of drought stress, and the expression of drought related genes increased, which may affect the ability of peanut plants to recover after drought.

Compared with the plants that experienced dehydration stress for the first time, the plants that repeatedly suffered dehydration stress and entered the water recovery stage alternately exhibited transcriptional and physiological memory responses during the subsequent dehydration stress [19,25]. For example, tobacco plants pretreated with methyl jasmonate showed “immune memory”, accumulating nicotine to cope with the second methyl jasmonate treatment [32].

Dehydration pretreatment increased the water holding capacity of the leaves of *Arabidopsis thaliana* [33] and maize [34]. In this study, we found that, in the leaf water content of Col, *atabcg22,* and *AhATL1-OX* during drought stress memory, the content of the second drought (D2 process) was similar to that of the first drought (D1 process), which was consistent with a previous report where the leaf relative water content was not affected by the previous drought stress [35], which may be related to the fact that the root system was not affected by drought training.

The proline contents of Col, *atabcg22,* and *AhATL1-OX* during drought training in the second drought (D2 process) were higher than in the first drought (D1 process), which was more conducive to maintaining the cell osmotic balance and resisting drought. We mainly focused on the comparison of proline content between Col and *P35S::AhATL1-eGFP* plants, and between *P35S::AhATL1-eGFP/atabcg22* and *atabcg22* plants. After second drought stress treatment (compared with the first drought stress treatment), the proline content of Col and *P35S::AhATL1-eGFP* plants were slightly increased. On the other hand, the proline content of *P35S::AhATL1-eGFP/atabcg22* was higher than that of *atabcg22*, and the proline content of *atabcg22* was higher than that of WT after first drought stress treatment. This may be because loss-of-function of *AtABCG22* might cause an accumulation of proline but less sensitivity to proline. This may be due to the regulation of proline by some other signaling or metabolic pathways, which made proline easier to accumulate or not easy to degrade. Overexpression of *AhATL1* can recover the loss of function of *AtABCG22*, resulting in insensitivity to proline. There is a high level of proline accumulation in the process of drought stress memory, and proline is a marker of drought degree, its metabolism and regulation mechanisms are relatively complex and proline may be under a positive feedback control. Proline accumulation is a significant metabolic adaptation mechanism of many organisms, including higher plants, under environmental stress, which is considered to play a protective role in plants under stress [36]. The proline content increased significantly after the first drought treatment and decreased with the rehydration treatment, reaching the second peak in the second recovery period and then remaining stable [37]. Regarding the ABA content of Col, *atabcg22,* and *AhATL1-OX* during drought training compared with the first drought (D1 process), the ABA contents of Col, *atabcg22,* and *AhATL1-OX* were increased during the second drought (D2 process), which was consistent with the reported changes in the endogenous ABA content in *Arabidopsis thaliana* [38].

The memory transcriptional pattern showed that the transcriptional behaviors of response genes under repeated stress were different from those under initial dehydration stress, indicating that stress memory is a complex phenotype, and is the result of multiple signal pathways [19,25,35]. Go analysis showed that [+/+] memory genes were involved in the ABA or abiotic stress response, [−/−] and [−/+] memory genes were involved in ribosome or protein synthesis, chloroplasts, and thylakoid membrane-related functions, and [+/−] memory genes were involved in a variety of signal pathway regulations, including the abscisic acid, ethylene, auxin, gibberellin, jasmonic acid, and salicylic acid pathway.

Transcriptional memory, similar to the activation of defense genes, can provide stronger or altered stress responses while reducing the consumption of readiness [18,26,36]. The overexpression of *AhATL1* changed from a [+/+] memory gene to a [−/+] memory gene in *Arabidopsis thaliana*. *AtRD29A*, *AtNCED3*, *AtSLAC1*, *AtALMT12,* and *AtABCG11* changed from [+/−] memory genes to [+/+] memory genes. *AtPP2C5*, *AtABCG40,* and *AtABCG25* changed from [+/−] memory genes to [−/+] memory genes. *AtUGT71C5* changed from a [−/+] memory gene to a [−/−] memory gene. *AtCYP707A1* changed from a post response gene type [=/+] to a memory gene type [−/+].

The [+/+] memory gene is a marker gene involved in the ABA or abiotic stress response [21]. The above results indicate that ABA induction and stress-response-related genes were not always [+/+] memory genes in Col, *atabcg22* mutants, or *AhATL1-OX* lines under drought training, and the mode transformation between memory genes might be more helpful for plant survival under repeated drought stress, especially in the case of the overexpression or knockout of a certain gene.

In summary, we found that the level of *AhATL1* expression and AhATL1 distribution increased more rapidly in D2 compared with in D1. Compared with the first recovery (R1), the *AhATL1* expression level and ABA content remained at a higher level during the second recovery (R2). The results show that the drought resistance ability of peanuts was enhanced after the first drought stress training. Overexpression of *AhATL1* in *Arabidopsis* Col lines was also investigated in this paper to further study the response mechanism of AhATL1. ABA induction and stress-response-related genes were not always [+/+] memory genes in Col, *atabcg22* mutants, or *AhATL1-OX* lines under drought training. The heterologous overexpression of *AhATL1* in *Arabidopsis* changed the expression pattern of certain memory genes and changed the post response gene type into a memory gene type, and the mode transformation between memory genes might be more helpful for plant survival under repeated drought stress, particularly in the case of the overexpression or knockout of a certain gene. The proline and water contents of AhATL1-OX plants during drought training demonstrated that the second drought (D2) was less severe than the first drought (D1), which was more conducive to maintaining cell osmotic balance and resisting drought. In the future, we will further study the molecular mechanism of its regulation from the perspective of the transcriptional level and epigenetics.

## 4. Material and Methods

### 4.1. Plant Material and Growth Conditions

**Figure 5:**
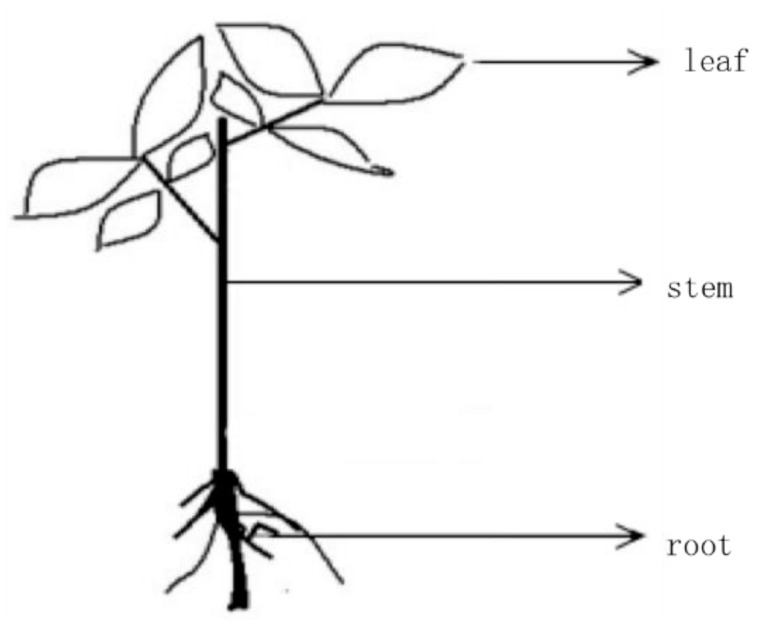
Schematic diagram of the peanut plant sampling.

Peanut cultivar ‘Yueyou 7′, which is a drought-resistant variety, was provided by the Crop Research Institute, Guangdong Academy of Agricultural Sciences. Put the peanuts (in a drying basin) in a light incubator (GuangQi, GHP-160, Shanghai, China) to dehydrate (28 °C, 48% RH, with a 16 h light and 8 h dark cycle, Light intensity13,000 lx) and treated for 1, 2, 4, 8, and 24 h in the light. At each sampling point, samples (0.5 g) of leaf (first functional leaf), stem (middle of the stem) and root (taproot, 1 cm) were excised as sample (Figure 5).

The peanut seedlings were dehydrated for 8 h (D1), and then rehydrated with 1/8 MS solution (pH 6.0) for 48 h. In the same way, the peanut plants were subjected to the second drought (D2) and then rehydrated (R2). Aerial tissue was harvested at noon on the day specified. The excised samples were immediately frozen and stored at −80 °C until use. All treatments were performed in at least three independent experiments.

**Figure 6:**
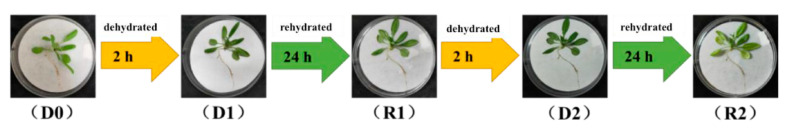
Schematic diagram of the *Arabidopsis* drought training.

Drought training of *Arabidopsis thaliana*: the *Arabidopsis* plant was taken out from the soil. The residual soil in the root was washed with water, then placed on the drying filter paper to dry for 2 h, and then put into a humid room. The rehydration was conducted by dripping water to the root for 24 h. The treatment of the subsequent air-drying and rehydration were the same as the first process (Figure 6). The leaves of *Arabidopsis thaliana* were collected and preserved in different periods [35].

### 4.2. Measurement of Endogenous ABA Content

From the frozen samples described in 4.1, the endogenous ABA was extracted. Measurement of endogenous ABA content as described by Hu et al. [16]. After grinding, extracting, freezing centrifugation and C18 filtration, samples were added to 96-well enzyme plates, with 50 mm^3^ per well. We added the first antibody, incubated at 37 ℃ for 0.5 h, washed the plate 4 times and dried it. Then, we added the second antibody, incubated at 37 ℃ for 0.5 h, washed the plate 4 times, then added the substrate for color development. When the color development was appropriate, we added 50 mm^3^ 2 mol/dm^3^ sulfuric acid to each well to terminate the reaction. The OD value of each sample at 490 nm was determined by enzyme-linked immunosorbent assay, and the content of ABA in the sample was calculated.

### 4.3. Immunofluorescence Localization Assays

Cryofixation and freeze-substitution of sample of leaf, stem and root in peanut were conducted as described by Kandasamy et al. [20]. Measurement of immunofluorescence localization assays was described by Hu et al. [16].

### 4.4. AhATL1 Overexpressing Arabidopsis Plants

*AhATL1* overexpression vector *P35S::AhATL1-eGFP* was transformed into Col wild type *Arabidopsis thaliana* by Agrobacterium Eha105. Eight positive strains were screened (Figure S1A, Supplementary Materials). All the positive transgenic seedlings were transplanted into soil for 3 weeks. DNA was extracted from rosette leaves and identified by PCR with primers AhATL1-ORF-F, AhATL1-ORF-R, and five positive strains were identified. The expression level of *AhATL1* gene was detected by RT-qPCR in five homozygous strains were screened by kanamycin resistance plate (Figure S1B). The results of RT-qPCR show that 5 strains were obtained. Among the positive transgenic plants, the expression level of *AhATL1* gene in line 3 was higher than that in other lines, so it was selected for further studies. As for transgenic lines, *P35S::AhATL1-eGFP/atabcg22* was obtained by crossing a wild-type *P35S::AhATL1-eGFP* line with *atabcg22* to screen homozygotes, so as to ensure the same genetic background and adequately compare their physiological differences. The plants were grown on peat-containing soil with a daily cycle of 16 h light and 8 h dark in well-watered conditions at 22 ± 2 °C and 60–70% relative humidity.

### 4.5. Measurement of Water Content

The seedlings were separated from the above ground part and the belowground part, and the fresh weight was weighed, respectively; after 72 h of drying in the oven, the dry weight was weighed to a constant weight. According to the water content = (fresh weight dry weight)/fresh weight, the water contents of the upper part and the belowground part were calculated, respectively.

### 4.6. Measurement of Proline Content

A series of standard curves of the proline solution were drawn. We accurately weighed 0.2 g of a mixed blade sample, added liquid nitrogen to grind into powder, and then quickly transferred the sample to a test tube. We added 2 cm^3^ 30 g/dm^3^ sulfosalicylic acid solution to each test tube, and the homogenate was extracted in a boiling water bath for 10 min after cooling. The sample was centrifuged at 3000× *g* for 10 min, and the supernatant was the extraction solution of proline.

We took 2 cm^3^ of supernatant into the tube with a plug, added 1 mL distilled water, 1 mL glacial acetic acid, and 2 cm^3^ acid ninhydrin reagent, and colored them in a boiling water bath for 1 h. After cooling, 2 cm^3^ toluene was added to each tube, and the tube was fully shaken for 30 s, and then placed for stratification. The upper toluene solution was absorbed into the colorimetric cup. Toluene was used as a blank control. The absorbance value was determined at 520 nm wavelength of the spectrophotometer (SHIMADZU, UV-1780, Tokyo, Japan). We determined the content of proline in the determination solution from the standard curve, and then calculated the percentage of the proline content in the sample.

### 4.7. Measurement of Malondialdehyde (MDA) Content

We accurately weighed 0.2 g of the blades, added liquid nitrogen to grind into powder, and then quickly moved the sample to the test tube. We added 2 cm^3^ 10% Trichloroacetic acid into the mixture, and the homogenate was centrifuged at 4000× *g* for 10 min. The supernatant was malondialdehyde extract. We took 1 cm^3^ of supernatant, added 1 cm^3^ of distilled water to the control tube, and then added 1 mL of 0.6 Barbituric acid solution to each tube. After shaking, the mixture was reacted in a boiling water bath for 15 min, and then centrifuged after rapid cooling. The absorbance of the supernatant was measured at 532 and 450 nm. The content of malondialdehyde was calculated by substituting it into the formula.

### 4.8. Expression Analysis

A SYBR premix ex taqtm II Kit (Takara Bio, Dalian, China) was used for real-time quantitative PCR. An ABI 7500 prism real-time PCR system (Applied Biosystems) was used. The PCR reaction conditions were as follows: 95 ℃ for 10 s, 1 cycle; 95 ℃ for 10 s, 62 ℃ for 10 s (depending on the optimal reaction temperatures of the different primers), 72 ℃ for 20 s, a total of 45 cycles; each sample was repeated three times, and finally the average value was taken. UBQ10 was used as an internal control. The melting curve was analyzed by reading the fluorescence intensity every 0.2 s between 62 and 95 ℃. The primers used in *Arabidopsis* qPCR as Table 2. The relative gene expression was calculated using 2-ΔΔCT methods (Table 2).

### 4.9. Statistical Analysis

The quantitative data are expressed as the means ± SD. The statistical significance of the experimental data was assessed using Student’s *t*-test or ANOVA (one-way analysis of variance with a least significant difference (LSD) post hoc test), as appropriate, using the SPSS17.0 statistical package (Chicago, IL, USA).

## Figures and Tables

**Table 1:** Classification of the ABA and stress response-related genes under drought training.

		Col	*abcg22*	*AhATL1-OX*
Memory gene	[+/+] D1-0h<D1-2h<D2-2h	*AtOST1*, *AtABI5*, *AtAREB1*, *AtBG2*	*AtOST1*, *AtABI5*, *AtAREB1*, *AtRD29A*,*AtNCED3*, *AtSLAC1*,*AtALMT12*, *AtABCG11*	*AtOST1*, *AtABI5*, *AtAREB1*, *AtRD29A*,*AtNCED3*, *AtSLAC1*,*AtALMT12*, *AtABCG11*
	[−/−] D1-0h>D1-2h>D2-2h			*AtUGT71C5*
	[+/−] D1-0h<D1-2h>D2-2h	*AtRD29A*,*AtNCED3*, *AtPP2C5*,*AtSLAC1*, *AtALMT12*, *AtABCG11*, *AtABCG22*,*AtABCG40*, *AtABCG25*		*AtABCG22*
Post response gene	[−/+] D1-0h>D1-2h<D2-2h	*AtUGT71C5*	*AtBG2*,*AtPP2C5*,*AtABCG40*, *AtABCG25*,*AtUGT71C5*, *AtCYP707A1*	*AtBG2*,*AtPP2C5*, *AtABCG40*,*AtABCG25*, *AtCYP707A1*
	[=/+] D1-0h=D1-2h<D2-2h	*AtCYP707A1*		
	[=/−] D1-0h=D1-2h>D2-2h			

The first symbol indicates that the transcriptional levels of the plants in D1-2h were higher (+), lower (−), or similar (=) compared with those in D1-0 h. The first symbol indicates that the transcriptional levels of the plants in D2-2h were higher (+), lower (−), or similar (=) compared with those in D1-2 h.

**Table 2:** The primers used in *Arabidopsis* qPCR.

Gene	Primer Sequence (5′→3′)
*AtActin*	F: GCTGTTGACTACGAGCAGGA
	R: TTCCATTCCCACAAACGAG
*AtRD29A*	F: CAAAGCAATGAGCATGAGCAAG
	R: CGGAAGACACGACAGGAAACAC
*AtNCED3*	F: AAGGTCGCAAGATTCGGGATT
	R: CGTTGAAAATTGAGTCTGGTGGAG
*AtCYP707A1*	F: TCTTCCAAACTCCCACTCCCT
	R: GCACGAACTTAGCAGCCTCTG
*AtUGT71C5*	F: CCGATGAAATAGCCACAGCC
	R: CCACCGTAGAAGACCCACCA
*AtBG2*	F: AAGAATGGATCACCGAGAAGGC
	R: TGGATGAAACAGTCCCCAAAACT
*AtPP2C5*	F: CGACCACCGATGCTTGACTT
	R: ACAACTTCCGCTCCTTTCTCC
*AtOST1*	F: TGGAGTTGCGAGATTGATGAGA
	R: AATGGCTAAATGGGTTGGTGTT
*AtABI5*	F: CACTTCCAGCTCCGCTTTGT
	R: GGTTGTCTAGCCGCAGTCTCA
*AtAREB1*	F: GTGTCGCCTGTTACGCCATT
	R: CGGTTCTTTATCATTCTCCTTTGC
*AtSLAC1*	F: GCGGGTTTGAATCAGGTGG
	R: TTTGCTGAGCGTTGATTTAGTCC
*AtALMT12*	F: TGAGCAAGACGAAGTGGATGG
	R: AGCAAAGAAACGAGTGTCAAGGA
*AtABCG11*	F: TTTGACAGCCAAGGGAGTGC
	R: TGATGAAGAAGATGATGCGGTAGA
*AtABCG22*	F: CGTGCGTGGTGTATCAGGTG
	R: ATGGTTCTAAGAGCAGTGGTGGA
*AtABCG40*	F: CCCAGGAAATGATAGAGCAAGG
	R: GGGTAACCGGAAATGGTGATG
*AtABCG25*	F: TAAACGCAGTCGCAGGGAGA
	R: TCCGAGGAAGACGAAGCAAA

## Supplementary Materials

The following are available online at https://www.mdpi.com/1422-0067/22/7/3398/s1, Figure S1: Filter of the transgenetic *Arabidopsis* in T1 generation and *AhATL1* relative expression level in *AhATL1* Overexpression Lines, Figure S2: Changes in the expression of *AtABCG22* gene under drought training.
