# Supplementary material for: Expression of AhATL1, an ABA Transport Factor Gene from Peanut, Is Affected by Altered Memory Gene Expression Patterns and Increased Tolerance to Drought Stress in Arabidopsis
Source: Int J Mol Sci. 2021 Mar 25;22(7):3398. doi: 10.3390/ijms22073398 (PMC8037416; doi:10.3390/ijms22073398)
Supplement: Supplementary file 1 [file ijms-22-03398-s001.pdf]

Supplementary Materials

AhATL1 overexpression vector *p35s:: AhATL1-eGFP* was transformed into Col wild type Arabidopsis thaliana by Agrobacterium Eha105. There were 8 positive strains were screened (supplementary Fig.S1A). All the positive transgenic seedlings were transplanted into soil for 3 weeks. DNA was extracted from rosette leaves and identified by PCR with primers AhATL1-ORF-F、AhATL1-ORF-R, and five positive strains were identified. The expression level of *AhATL1* gene was detected by RT-qPCR in five homozygous strains were screened by resistance plate (supplementary Fig.S1B). The results of RT-qPCR showed that 5 strains were obtained. Among the positive transgenic plants, the expression level of *AhATL1* gene in line 3 was higher than that in other lines, so the selection was more suitable. The No.3 overexpression line (*AhATL1-ox*) was selected for follow-up experiment.

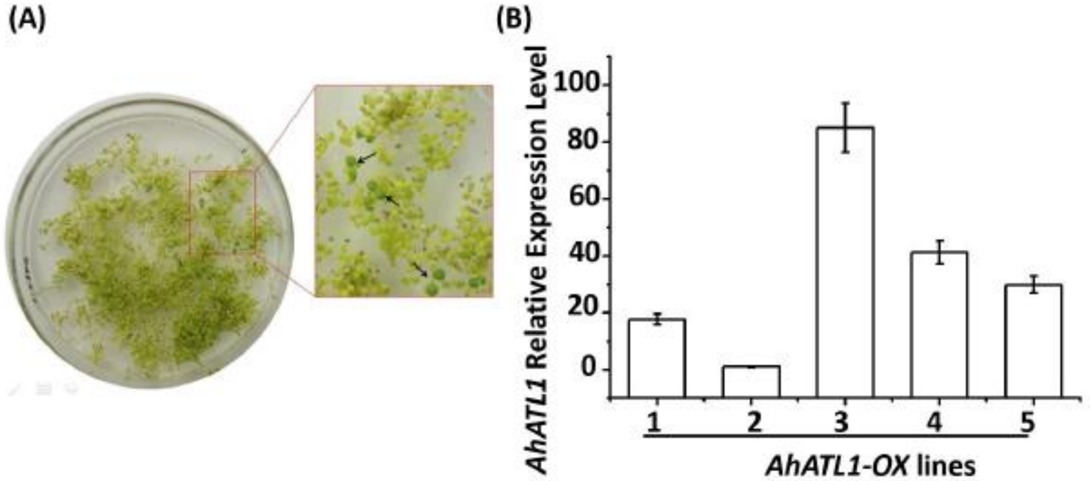

Supplementary Fig. S1: Filter of the transgenetic *Arabidopsis* in T1 generation and *AhATL1* relative expression level in *AhATL1* Overexpression Lines

(A) The T1 generation plants of *AhATL1* transgenic Arabidopsis were screened by Kana resistance plate. The black tip indicated the positive transgenic seedlings with Kana resistance

(B) Detection of the expression of *AhATL1* in positive transgenic *Arabidopsis* thaliana lines by RT qPCR

The results of qPCR showed that the mutant *atabcg22* had no *AtABCG22* gene expression in the four stages of stress memory, while wild-type Col and *AhATL1-OX lines* had *AtABCG22* gene expression (supplementary Fig.S2).

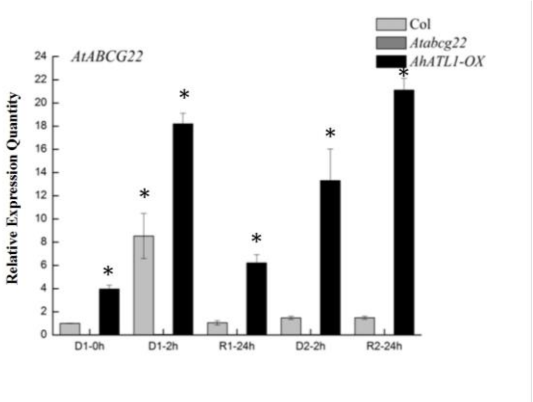

Supplementary Fig. S2 Changes in the expression of *AtABCG22* gene under drought training
